# Cardioform Septal Occluder for the Closure of a Peri-Device Leak After Initial Watchman Implantation

**DOI:** 10.7759/cureus.15439

**Published:** 2021-06-04

**Authors:** Jose R Sleiman, Antonio J Lewis, Eduardo J Perez, Craig R Asher, Roberto J Cubeddu

**Affiliations:** 1 Cardiology, Cleveland Clinic Florida, Weston, USA

**Keywords:** left atrial appendage closure, peri-device leak, cardioform septal occluder, watchman, residual leak, atrial fibrillation, stroke, oral anticoagulation

## Abstract

Left atrial appendage occlusion has become a safe and effective alternative for stroke-risk reduction among patients with non-valvular atrial fibrillation (AF). Although complete closure is ideal, residual peri-device leaks (PDL) are not uncommon and have been associated with an increased residual risk of stroke. PDL closure has been proposed as an alternate strategy to allow for the safe discontinuation of oral anticoagulation. We describe the safety and feasibility of successful PDL closure using a non-fenestrated Cardioform (Gore Medical, Flagstaff, Arizona) septal occluder after initial Watchman (Boston Scientific, Marlborough, Massachusetts) implantation.

## Introduction

Percutaneous left atrial appendage occlusion (LAAO) has evolved as a safe and effective therapy for stroke risk reduction in patients with non-valvular atrial fibrillation (AF)[1]._ _Presently, the Watchman occluder (Boston Scientific, Marlborough, Massachusetts) remains the only Food and Drug Administration (FDA) approved device in the United States for this indication. While successful in most cases, incomplete closure, often recognized as a peri-device leak (PDL), can often be seen. Despite improvements with Watchman closure techniques, the presence of a significant PDL, defined as a residual gap of >5 mm, may be expected in up to 5% of patients [1-2]. Similar findings have been reported with other novel LAAO devices. In these patients, discontinuation of oral anticoagulation (OAC) is felt unsafe and is associated with increased stroke risk. The practice of PDL closure has been proposed and increasingly adopted as an alternative strategy with promising results [3-4]. We report the novel use of a Cardioform Septal Occluder (CSO) (Gore Medical, Flagstaff, Arizona) for PDL closure following the initial implantation of a Watchman device in a patient with a complex multilobar LAA.

## Case presentation

**Figure 1:**
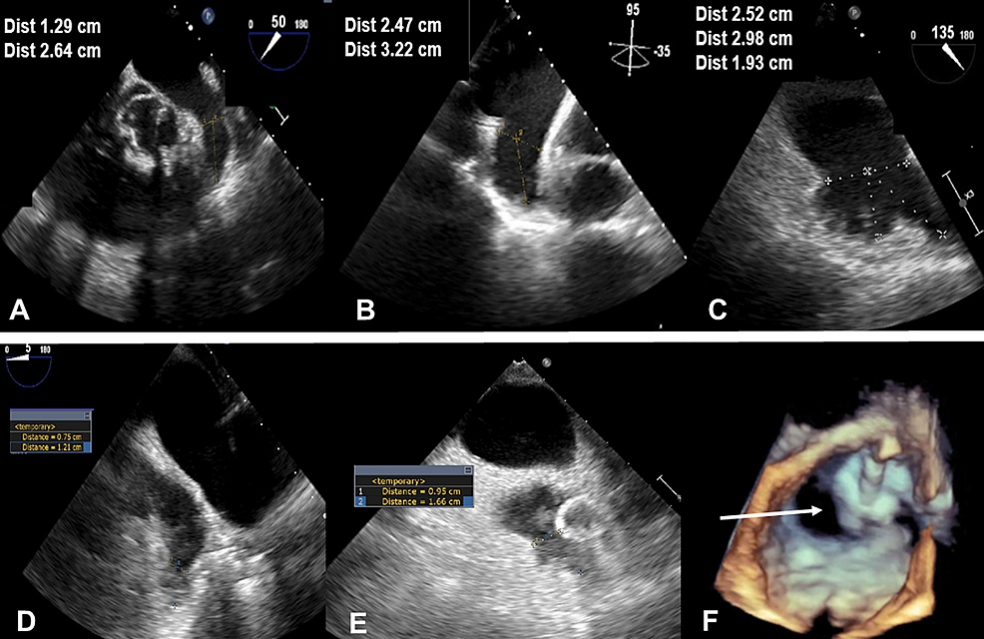
Multiplane transesophageal echocardiography during left atrial appendage closure Panels A-C: Multiplane TEE showing large bilobar LAA. Panel B: Demonstrates the longest depth measuring 3.22 cm. Panel C: Demonstrates the widest ostial diameter of 2.52 cm. Panels D-E: Show a moderate PDL size involving the posterior lobe measuring 9 mm in width by 16 mm in length. Panel F: 3D TEE showing moderate size PDL (arrow) in proximity to the Watchman occluder. TEE: transesophageal echocardiography; LAA: left atrial appendage; PDL: peri-device leak

**Figure 2:**
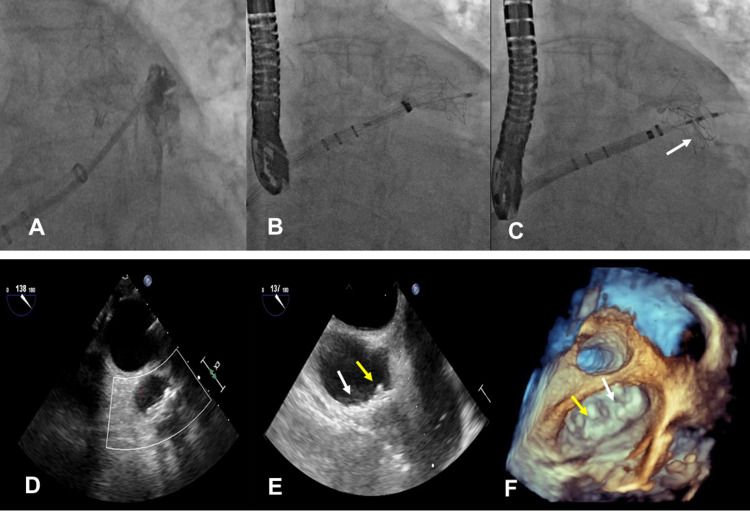
Percutaneous closure of moderate peri-device leak with Cardioform septal occluder (CSO) after initial Watchman implant Panel A: Angiographic examination of PDL in proximity to the Watchman occluder. Panels B-C: Fluoroscopic image showing delivery of the 25 mm Cardioform septal occluder within the PDL (arrow). Panels D-F: TEE showing closure of LAA and PDL with the Watchman occluder (yellow arrow) and CSO (white arrow), respectively. Panel F: 3D TEE en face showing both occluders. TEE: transesophageal echocardiography; LAA: left atrial appendage; PDL: peri-device leak

An 80-year-old man with non-valvular AF was referred for LAAO. Baseline transesophageal echocardiography (TEE) revealed a large LAA, with a bilobar broccoli configuration, maximal ostial width of 25.2 mm, and anterior lobe depth of 32.2 mm (Figures 1A-1C). The posterior lobe, while sharing the same ostium, was shallow and measured 19.3 mm in depth. LAAO was attempted under general anesthesia through a right transfemoral venous approach. Transseptal puncture was performed along the inferior-posterior septal wall using fluoroscopic and intraoperative TEE guidance. Weight-based intravenous heparin was administered to maintain ACT levels >250-300 seconds throughout the procedure. The transseptal sheath was exchanged over a 0.35 extra-stiff Amplatz wire (Cook Medical, Bloomington, Indiana) for a 14 Fr anterior curved Watchman delivery guide catheter. LA angiography with a 6 Fr pigtail catheter provided further clarity of LAA morphology. Baseline measurements dictated the use of a 30 mm Watchman occluder. A 14 Fr Boston Scientific anterior curved catheter (Boston Scientific, Marlborough, Massachusetts) was used to deliver the occluder within the anterior lobe of the LAA. The tug test ensured device stability. Initial deployment resulted in satisfactory landing depth, adequate ostial alignment, and device compression (range 18% to 23%). A residual PDL measuring 9 x 16 mm located within the posterior lobe (Figures 1D-1F) was detected. The options to recapture, reposition, and/or exchange for a larger occluder were considered unlikely to be beneficial. Watchman followed by ad hoc PDL closure was considered. The Watchman occluder was released within the anterior lobe, and PDL closure pursued with a 25 mm CSO. Access to the residual PDL was obtained through the 14 Fr Watchman delivery catheter using a standard 0.035 soft J-wire over a 5 Fr multipurpose catheter that was subsequently exchanged for a super-stiff Amplatz wire. The distal disc of the CSO was deployed within the PDL sac and the proximal disc on the left atrial side to allow for inter-digitization of the Watchman occluder, thus minimizing the risk of device migration or residual leak (Figures 2A-2C). The device was released following confirmation of device stability and any residual leak on TEE (Figures 2D-2F). The procedure was uneventful, and the patient was discharged home on OAC and daily aspirin. OAC was discontinued 45 days later after TEE confirmed the absence of any residual leak, device-related thrombus, or migration. The patient was maintained on dual antiplatelet therapy with clopidogrel and aspirin for an additional six months, after which clopidogrel was discontinued. No neurological events were reported at the interim follow-up of 18 months.

## Discussion

The clinical management of PDL following initial LAAO remains poorly understood. While small PDLs appear to be clinically irrelevant, in larger defects (PDL size ≥ 5 mm), the discontinuation of OAC is generally unadvisable and believed to be associated with increased stroke risk [4]. For patients in whom the continued use of OAC represents a major risk, PDL closure has been proposed as a safe and alternative strategy.

In a previous systematic review of PDL closure following incomplete LAAO by Sleiman et al., the safety and feasibility of PDL closure are underscored and associated with high procedural success (90%) and low procedural adverse event rates (2.8%), with no stroke events reported at a mean follow-up of six months [5]. Importantly, the cumulative experience not only highlights the lack of consensus when it comes to device selection but also the large spectrum of PDL size and shapes and the value of ensuring access and the technical dexterity to a myriad of endovascular occluders or plugs that may be safe when tailored to the anatomical characteristics of the defect [5].

In this report, we describe the novel use of the CSO to treat a residual PDL following initial Watchman implantation, adding to the cumulative experience of PDL closure. The safety and feasibility of the CSO to close a moderate-to-large 9 x 16 mm residual PDL in an underlying complex bilobar appendage is exemplified. The soft, conformable, non-fenestrated design of the CSO resulted in successful PDL closure, with no evidence of device migration, leak, or thrombus during the six-month follow-up examination, allowing the patient to safely discontinue OAC.

The presence of a PDL or a residual LAA gap following endovascular or surgical LAA closure remains a common problem. While the residual stroke risk associated with these defects remains poorly understood, multiple efforts have been made to determine the predictors and characteristics of these defects [3]. Until further clarification, thromboembolic protection with OAC is recommended in patients with large PDL. For many patients at risk of complications related to long-term OAC, this strategy offers its own limitations. Percutaneous closure of PDL has evolved as an alternate solution for these patients [3-5].

The percutaneous approach to the closure of PDL is well-described. Based on the published data, the management of smaller and moderate-sized defects is usually accomplished by the use of endovascular coils and endovascular plugs, and second LAAO devices are more commonly reserved for larger leaks [6-10]. There are multiple reports of the use of different endovascular plugs; the most commonly employed being the Amplatzer vascular plug (St. Jude Medical, Inc., St. Paul, MN) [5,7,10] and Amplatzer septal occluders (St. Jude Medical) [11]. Recent data showed the use of CSO as a second occluder device for post-surgical and post-Lariat LAAO-related leaks [12]. However, to our knowledge, this is the first report using a CSO to close a PDL following a Watchman implant. The residual PDL (9 mm) was successfully closed ad-hoc using a 1:2.5 ratio with a 25 mm CSO after initial LAA implantation of a 30 mm Watchman occluder.

CSO is a non-fenestrated endovascular plug that is available in three different sizes (20, 25, and 30 mm) and approved for the closure of interatrial septal defects. Similar to other off-label indications [13], the CSO adequately conformed within the residual leak gap given its ability to provide unique conformability and adapt to a broad variability morphology. Device selection was based on a) the size and shape of the PDL and b) the soft-atraumatic, non-fenestrated, and conformable nature of the CSO. A straddling technique was employed across the free shoulder edge of the Watchman occluder to ensure adequate closure while minimizing the risk of both embolization and residual leak. Finally, our case supports the safety and feasibility of the use of the CSO for patients with PDL following a Watchman implant. PDL closure allowed for the safe discontinuation of OAC in our patient. TEE examination at 45 days confirmed the stability of both the Watchman and CSO and the absence of any residual leak or device-related thrombus. After 18 months, the patient remains asymptomatic and free of neurological events on daily aspirin only.

The mechanisms of PDL, while multiple, most commonly result from a suboptimal device implantation technique and the large variability of LAA size and morphology. A multitude of novel surgical and endovascular device iterations and techniques are being developed to overcome the existing limitations of incomplete LAAO.

## Conclusions

When anatomically suitable, the CSO may be added to the list of alternative devices that may be considered for the closure of residual peri-device leaks following LAAO.
